# Tumor Marker-Based Definition of the Transarterial Chemoembolization-Refractoriness in Intermediate-Stage Hepatocellular Carcinoma: A Multi-Cohort Study

**DOI:** 10.3390/cancers11111721

**Published:** 2019-11-04

**Authors:** Jun Sik Yoon, Dong Hyun Sinn, Jeong-Hoon Lee, Hwi Young Kim, Cheol-Hyung Lee, Sun Woong Kim, Hyo Young Lee, Joon Yeul Nam, Young Chang, Yun Bin Lee, Eun Ju Cho, Su Jong Yu, Hyo-Cheol Kim, Jin Wook Chung, Yoon Jun Kim, Jung-Hwan Yoon

**Affiliations:** 1 Department of Internal Medicine and Liver Research Institute, Seoul National University College of Medicine, Seoul 03080, Korea; yojusi@naver.com (J.S.Y.); hyung6519@gmail.com (C.-H.L.); withtwohands@gmail.com (S.W.K.); catchhyong@gmail.com (H.Y.L.); moreno777@gmail.com (J.Y.N.); chyoung86@gmail.com (Y.C.); yblee@snu.ac.kr (Y.B.L.); creatioex@gmail.com (E.J.C.); ydoctor2@hanmail.net (S.J.Y.); yoonjun@snu.ac.kr (Y.J.K.); yoonjh@snu.ac.kr (J.-H.Y.); 2 Department of Internal Medicine, Busan Paik Hospital, Inje University College of Medicine, Busan 47392, Korea; 3 Department of Internal Medicine, Samsung Medical Center, Seoul 06351, Korea; sinndhn@hanmail.net; 4 Department of Internal Medicine, College of Medicine, Ewha Womans University, Seoul 07804, Korea; 5 Department of Internal Medicine, Eulji General Hospital, Eulji University School of Medicine, Seoul 01830, Korea; 6 Department of Internal Medicine, Digestive Disease Center, Institute for Digestive Research, Soonchunhyang University College of Medicine, Seoul 04401, Korea; 7 Department of Radiology, Seoul National University College of Medicine, Seoul 03080, Korea; angiointervention@gmail.com (H.-C.K.);

**Keywords:** tumor marker, alpha-fetoprotein, protein induced by vitamin K absence-II, hepatocellular carcinoma, tumor biology

## Abstract

Background: For patients with hepatocellular carcinoma (HCC), the definition of refractoriness to transarterial chemoembolization (TACE), which might make them a candidate for systemic therapy, is still controversial. We aimed to derive and validate a tumor marker-based algorithm to define the refractoriness to TACE in patients with intermediate-stage HCC. Methods: This multi-cohort study was comprised of patients who underwent TACE for treatment-naïve intermediate-stage HCC. We derived a prediction model for overall survival (OS) using the pre- and post-TACE model to predict tumor recurrence after living donor liver transplantation (MoRAL) (i.e., MoRAL score = 11×√protein induced by vitamin K absence-II + 2×√alpha-fetoprotein), which was proven to reflect both tumor burden and biologic aggressiveness of HCC in the explant liver, from a training cohort (n = 193). These results were externally validated in both an independent hospital cohort (from two large-volume centers, n = 140) and a Korean National Cancer Registry sample cohort (n = 149). Results: The changes in MoRAL score (ΔMoRAL) after initial TACE was an independent predictor of OS (MoRAL-increase vs. MoRAL-non-increase: adjusted hazard ratio (HR) = 2.18, 95% confidence interval (CI) = 1.37–3.46, *p* = 0.001; median OS = 18.8 vs. 37.8 months). In a subgroup of patients with a high baseline MoRAL score (≥89.5, 25th percentile and higher), the prognostic impact of ΔMoRAL was more pronounced (MoRAL-increase vs. MoRAL-non-increase: HR = 3.68, 95% CI = 1.54–8.76, *p* < 0.001; median OS = 9.9 vs. 37.4 months). These results were reproduced in the external validation cohorts. Conclusion: The ΔMoRAL after the first TACE, a simple and objective index, provides refined prognostication for patients with intermediate-stage HCC. Proceeding to a second TACE may not provide additional survival benefits in cases of a MoRAL-increase after the first TACE in patients with a high baseline MoRAL score (≥89.5), who might be candidates for systemic therapy.

## 1. Introduction

Hepatocellular carcinoma (HCC) is an aggressive tumor that is the fourth leading cause of cancer-related mortality worldwide [1]. In patients with intermediate-stage (Barcelona Clinic Liver Cancer [BCLC] stage B) HCC, who have multinodular HCC without macrovascular invasion or extrahepatic metastasis, the recommended treatment modality is transarterial chemoembolization (TACE) [2,3]. TACE is performed in a palliative manner, and the decision-making for retreatment with TACE is critical in managing HCC patients. The decision to repeat TACE or switch to other treatment modalities should be determined on the basis of the clinical response of the HCC patient. In cases of TACE refractoriness, the switch from TACE to sorafenib has been reported to improve survival in intermediate-stage HCC patients [4,5,6].

There have been several decision-making models developed for TACE retreatment, such as the Assessment for Retreatment with TACE (ART) score [7] and the ABCR (standing for alpha-fetoprotein (AFP), BCLC, Child-Pugh, and Response) score [8], which have been controversial in their efficacy in retrospective validation studies [9,10,11,12]. Therefore, such scoring systems for TACE retreatment were not adopted in the most recent practice guidelines [13,14]. Moreover, the scoring systems (i.e., the ART score and ABCR score) have shown that radiological tumor response plays a key role in this clinical decision. Although the radiological tumor response has a strong prognostic performance after TACE [15,16,17,18], the interpretation of the radiological tumor response is complex and heavily depends on the individual radiologist’s subjective measurement. As a result, inter-reader disagreement might occur in the radiological tumor response [19,20]. Therefore, an objective and reproducible model that can help decision-making for retreatment with TACE is warranted.

Recently, we developed a model to predict tumor recurrence after living donor liver transplantation (LT) (i.e., a model to predict tumor recurrence after living donor liver transplantation (MoRAL score)) using only two HCC-related tumor markers: protein induced by vitamin K absence-II (PIVKA-II) and AFP [21]. The histological analysis of the explant liver showed that the MoRAL score had significant positive correlations (positive Pearson correlation coefficients) with histological prognostic factors (i.e., Edmonson-Steiner nuclear grade, microvascular invasion, perineural invasion, etc.). Moreover, the MoRAL score was also correlated with entire tumor volume (i.e., size and number). The MoRAL score is an objective and reproducible model that can reflect both tumor burden and biological aggressiveness. We hypothesized that an algorithm utilizing the MoRAL score could also provide refined prognostication in patients with intermediate-stage HCC who have a high tumor burden and aggressive tumor biology.

Therefore, in this study, we aimed to derive and validate the MoRAL score-based algorithm for guiding the decision for retreatment with TACE in patients with intermediate-stage HCC.

## 2. Results

### 2.1. Clinical Characteristics

During the study period, among the 745 patients who were admitted to SNUH with treatment-naïve intermediate-stage HCC, 290 patients were eligible for this study and 97 patients were excluded according to exclusion criteria (Supplementary Figure S1A). Finally, a total of 193 patients were included as the training cohort. The same inclusion/exclusion criteria were applied in the two other hospitals during the study period and 140 patients were included as the hospital validation cohort (Supplementary Figure S1B). In the Korean Primary Liver Cancer Registry database, among the 10,578 patients who had treatment-naïve HCC, 637 patients were intermediate-stage HCC and were treated with TACE as a first treatment. Among them, 149 patients were included as the nationwide validation cohort (Supplementary Figure S1C).

The characteristics of the three cohorts prior to the first and second TACE, and changes in the prognostic variables, are shown in Table 1. Viral hepatitis was the most common cause of HCC. The majority of patients with chronic hepatitis B in the training cohort (125/132, 94.7%) and hospital validation cohort (90/99, 90.9%) were treated with nucleos(t)ide analogues, and the rates were not significantly different between the two cohorts (*p* = 0.26 by Chi-square test). The rates of sustained viral response in patients with chronic hepatitis C were also not significantly different between the two cohorts (25.0% vs. 33.3%; *p* = 0.70 by Fisher’s exact test). In the training cohort (n = 193), the median MoRAL score decreased from a baseline of 259.2 (IQR = 89.8–778.2) to 93.8 (IQR = 60.9–231.4). Most patients were classified as MoRAL-non-increase and 33 patients (17.1%) were classified as MoRAL-increase between the first and second TACE sessions. The median and mean time intervals between the first and second TACE were 55 days (interquartile range (IQR) = 41.5–70.0 days) and 57.2 days (95% confidence interval (CI), 54.4–60.1 days), respectively. The median total number of TACE sessions was six (IQR = 4.0–8.0).

In the hospital validation cohort (n = 140), the baseline MoRAL score (237.8, IQR = 93.1–419.3) was lower than in the training cohort. Between the first and second TACE, 41 patients (29.3%) were classified as MoRAL-increase. The median and mean time intervals between the first and second TACE were 35 days (IQR = 30.0–43.0 days) and 39.9 days (95% CI, 37.5–42.3 days), respectively. The median total number of TACE sessions was five (IQR = 2.3–8.0). In the nationwide validation cohort (n = 149), the baseline MoRAL score (311.9, IQR = 133.0–544.6) was the highest among the three cohorts. The data of tumor markers before the second session of TACE were not available from the database. The median and mean time intervals between the first and second TACE were 50 days (IQR = 34.0–62.0 days) and 50.1 days (95% CI, 47.2–53.0 days), respectively.

### 2.2. Derivation of Prognostic Models from the Training Cohort

**Figure 1:**
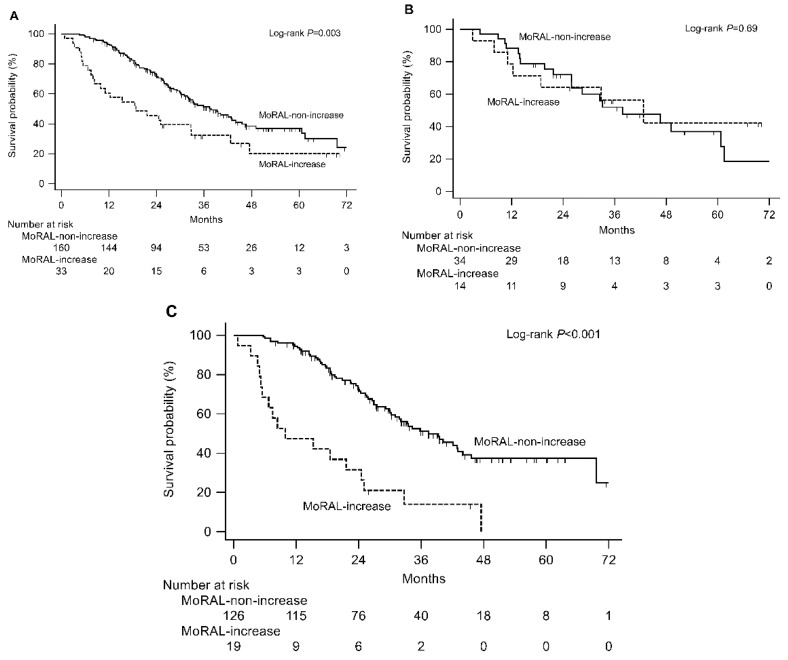
Prognostic significance of ΔMoRAL among the entire study population (**A**) and patients baseline MoRAL score ≤89.5 (**B**) and >89.5 (**C**) in the training cohort.

In the training cohort, 106 patients (54.9%) died during the study period (median follow-up duration = 47.4 months, IQR = 31.8–62.5 months) and the median OS was 26.1 months (IQR = 16.0–40.0 months). Among the baseline and post-TACE variables, the baseline Child-Pugh class, radiological tumor response, worsening in Child-Pugh score between the first and second sessions of TACE, and ΔMoRAL had significant associations with OS in a univariable analysis (all log-rank *p* < 0.05, Table 2). The risk of death was significantly higher in patients with a MoRAL-increase than in those with a MoRAL-non-increase (hazard ratio (HR) = 1.96, 95% CI = 1.24–3.09, *p* = 0.003). The median OS was 18.8 months (95% CI = 9.9–32.8 months) in the MoRAL-increase patients and 37.8 months (95% CI = 31.8–45.6 months) in the MoRAL-non-increase patients (Figure 1A). However, the baseline MoRAL score and serum levels of the respective tumor markers (i.e., AFP and PIVKA-II) had no significant associations with OS. There was a significant correlation between each radiological tumor response (PR, SD, or PD) and the ΔMoRAL percentage (Spearman *r* = 0.23, *p* = 0.001; Supplementary Figure S2). To avoid statistical redundancy, we performed a multivariable analysis separately using the radiological tumor response (model 1) and ΔMoRAL (model 2), along with the significant predictive variables from the univariable analysis. In model 1, the adjusted HR (aHR) of the absence of radiological PR was 1.63 (95% CI = 1.08–2.46, *p* = 0.019; Table 3). In model 2, the aHR of MoRAL-increase was 2.18 (95% CI = 1.37–3.46, *p* = 0.001). Although there was no statistically significant difference, patients with alcoholic liver disease tended to have a shorter median OS than patients with other causes (25.3 months vs. 39.3 months, *p* = 0.10). Therefore, in order to avoid possible bias from including patients with alcoholic liver disease, we conducted a Cox regression analysis in a subgroup excluding 20 patients with alcoholic liver disease. In the subgroup analysis, the aHR of MoRAL-increase was 2.06 (95% CI, 1.27–3.35, *p* = 0.004), which was similar to that of the entire population (Supplementary Table S1).

We divided patients in the training cohort using a cutoff of the 25th percentile value of the baseline MoRAL score (i.e., 89.5). In patients with a low baseline MoRAL score (<89.5), ΔMoRAL was not associated with OS (HR = 0.84, 95% CI = 0.37–1.92, *p* = 0.69; Figure 1B). However, in patients with a high baseline MoRAL score (≥89.5), the prognostic impact of ΔMoRAL was more pronounced than in the whole study population (MoRAL-increase vs. MoRAL-non-increase: HR = 3.68; 95% CI = 1.54–8.76, *p* < 0.001). The median OS times of the MoRAL-increase patients and MoRAL-non-increase patients were 9.9 months (95% CI = 5.5–24.5 months) and 37.4 months (95% CI = 31.0–44.0 months), respectively (Figure 1C).

### 2.3. External Validation in the Hospital and Nationwide Validation Cohorts

**Figure 2:**
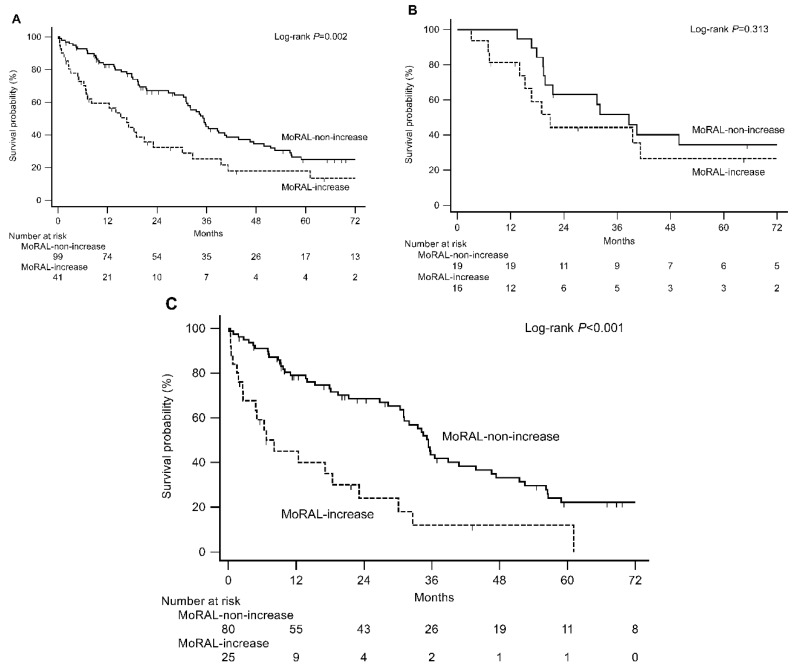
Prognostic significance of ΔMoRAL among the entire study population (**A**) and patients with baseline MoRAL score ≤89.5 (**B**) and >89.5 (**C**) in the hospital validation cohort.

In the hospital validation cohort, 93 patients (66.4%) died during the study period (median follow-up duration = 86.6 months (IQR = 72.6–105.9)) and the median OS was 21.1 months (IQR = 8.9–41.0 months). In the nationwide validation cohort, 105 patients (70.5%) died during the study period (median follow-up duration = 63.7 months, IQR = 40.5–82.2 months) and the median OS was 26.0 months (IQR = 14.3–42.3 months). The baseline MoRAL score was not associated with OS in both the hospital and nationwide validation cohorts (Supplementary Figure S3). In the hospital validation cohort, the OS of the patients with MoRAL-increase was significantly shorter than that of patients with MoRAL-non-increase (HR = 1.99, 95% CI = 1.28–3.09, *p* = 0.002). The median OS times of MoRAL-increase and MoRAL-non-increase patients were 16.7 months (95% CI = 7.2–23.1 months) and 35.4 months (95% CI = 31.1–40.8 months), respectively (Figure 2A). We also divided patients in the hospital validation cohort according to baseline MoRAL score with a cutoff value of 89.5. The ΔMoRAL was not associated with OS (HR = 1.53, 95% CI = 0.64–3.67, *p* = 0.313) in patients with a low baseline MoRAL score (<89.5, Figure 2B). However, ΔMoRAL showed a strong predictive performance for OS in patients with a high baseline MoRAL score (≥89.5) (MoRAL-increase vs. MoRAL-non-increase: HR = 2.90, 95% CI = 1.41–5.95, *p* < 0.001). The median OS was 8.1 months (95% CI = 2.6–18.4 months) in patients with MoRAL-increase and 35.7 months (95% CI = 31.0–43.8 months) in those with MoRAL-non-increase (Figure 2C).

### 2.4. Comparison with Other Indices

As shown in Table 4, the performance of ΔMoRAL in predicting OS was significantly better than the performances of initial respective serum tumor markers, baseline MoRAL score, and changes in serum tumor markers, except for the baseline AFP level, in both the training and hospital validation cohorts (all *p* < 0.05). The ΔMoRAL showed a higher *c*-index than the baseline serum AFP level in the hospital validation cohort, without statistical significance (*p* = 0.17). The ΔMoRAL also showed a higher *c*-index than the radiological tumor response and ART score of 2.5 in both cohorts, albeit without statistical significance (all *p* > 0.05).

## 3. Discussion

In this study, we tried to find an algorithm to guide decision-making for retreatment with TACE in patients with intermediate-stage HCC using the MoRAL score. We derived and validated the MoRAL score-based algorithm using data from three large-volume hospitals and a nationwide cancer sampling registry in Korea. Although the baseline MoRAL score did not show a prognostic impact on OS, ΔMoRAL was able to differentiate two prognostic subgroups. Moreover, the prognostic impact of ΔMoRAL was more pronounced in patients with a high baseline MoRAL score (≥89.5). Patients with a high baseline MoRAL score (≥89.5) and MoRAL-increase between the first and second TACE sessions had a dismal prognosis (median OS <10 months) in both the training and validation cohorts.

The ART score [7] and ABCR score [8] are the most popular models currently to guide decision-making for retreatment with TACE. The ART score is calculated on the basis of three parameters (increase in Child-Pugh score, increase in aspartate aminotransferase by >25% from baseline, and tumor response). The ART score is calculated before performing a second TACE, and, on the basis of this score, patients can be differentiated into two groups, each with a distinct prognosis. For patients with an ART score of 2.5 or higher, further TACE sessions may not provide additional survival benefit and switching to sorafenib should be considered. The ABCR score was proposed by a French group. The ABCR score includes four parameters (BCLC stage and AFP at baseline, increase in Child-Pugh score, and absence of radiologic response). Patients with an ABCR score of four or higher prior to the second TACE may not benefit from further TACE sessions. However, these scoring systems have not been validated in retrospective validation studies [9,10,11,12] and may be vulnerable to bias regarding the subjective measurement of the radiologic tumor response [19,20].

The MoRAL score is calculated from serum AFP and PIVKA-II, and shows refined prognostication for tumor recurrence after LT. HCC patients with high serum AFP (>400 ng/mL) had a poor outcome and showed significant upregulation of signaling pathways—not only VEGF but also IGF1R, NOTCH, and mTOR [22]. Serum PIVKA-II has been reported to promote HCC invasion by promoting epithelial-mesenchymal transition [23]. The MoRAL score was deemed to reflect tumor biology and stemness precisely because it accurately predicts tumor recurrence after eradication of all intrahepatic visible tumors when using immunosuppressive agents, which could facilitate tumor regrowth [24]. Moreover, a higher MoRAL score was well correlated with histological findings associated with worse prognosis, such as high Edmonson-Steiner nuclear grade, microvascular invasion, bile duct invasion, perineural invasion, and serosal invasion [21]. In addition, a high MoRAL score was associated with cytokeratin 19 expression, which is a stemness-related marker and reflects tumor invasiveness [25,26,27], among patients who underwent surgical resection for HCC [28]. Therefore, we attempted to verify the usefulness of the original MoRAL score, assuming that the baseline MoRAL score would be helpful as well in the decision-making process for retreatment with TACE. However, unlike the HCC patients receiving LT, which is a curative treatment option, the baseline MoRAL score was not associated with OS in HCC patients receiving TACE in the present study. Moreover, variables of baseline tumor characteristics such as size and number of tumors were not associated with OS, which was consistent with the findings of two previous studies from which the ART and ABCR scores were derived [7,8]. These results indicated that the prognosis of patients with intermediate-stage HCC undergoing TACE relies more significantly on the treatment response, rather than the baseline tumor characteristics. In our study, the treatment response after TACE was investigated through both ΔMoRAL and the radiological tumor response. The ΔMoRAL score had a significant correlation with radiological tumor response, showing a higher *c*-index than the radiological tumor response.

The prognostic impact of ΔMoRAL was more pronounced in patients with a high baseline MoRAL score (≥89.5, 25th percentile and higher). We used a different cutoff value in the current study than was used in the previous study (314.8 in the original MoRAL study by Lee et al.) [21], since the potency of treatment modalities (TACE vs. LT) is quite different. Although the baseline MoRAL score itself was not a predictor of OS, it was useful for detecting patients with poor expected OS in conjunction with ΔMoRAL. For patients with a high baseline MoRAL score (≥89.5) followed by MoRAL-increase between the first and second TACE sessions, the expected OS was shorter than 10 months despite further TACE sessions. The OS of the patients in this group was as poor as the OS of the BCLC B control (placebo) group in the Sorafenib Hepatocellular Carcinoma Assessment Randomized Protocol trial (11.4 months) [4]. This short-expected OS implied that further TACE sessions may not provide an additional survival benefit in this group. Therefore, switching to other evidence-based systemic treatments such as sorafenib therapy should be considered in this group of patients [4,5,6]. Moreover, immune checkpoint inhibitors with a promising treatment response in advanced-stage HCC, such as nivolumab [29] and pembrolizumab [30], might be applied in this group of patients (i.e., baseline MoRAL score of ≥89.5 with MoRAL-increase).

There are several limitations to the present study. First, because of the retrospective nature of this study, it was prone to selection bias. Approximately one-fifth of the patients satisfying the inclusion criteria were excluded because of incomplete laboratory results for tumor markers in the training cohort. However, we have shown that the performance of the MoRAL score-based algorithm was reproducible in the independent external validation cohort. Second, because Korea is a hepatitis B-endemic area [31], approximately 70% of the cases of HCC in our study population were caused by hepatitis B virus infection. Our results need to be further validated in areas with different underlying etiologies of HCC worldwide, especially in Western countries where hepatitis C virus is not a major cause of HCC [32]. Third, in patients with a low baseline MoRAL score (≤89.5), ΔMoRAL did not predict OS. However, the median OS of patients with a low baseline MoRAL score (≤89.5) in the training cohort, hospital validation cohort, and nationwide validation cohort were 42.7, 32.1, and 38.0 months, respectively, which indicated good prognoses. Therefore, further TACE sessions could be recommended regardless of ΔMoRAL in these subgroups. Fourth, our prediction model did not reflect liver function, such as the baseline Child-Pugh class or Child-Pugh increase. Due to the different characteristics and dynamics between the two cohorts, prediction models that reflect both liver function and ΔMoRAL in the training cohort were not validated in the hospital validation cohort. However, ΔMoRAL consistently showed good predictive performance of OS in both cohorts. Moreover, ΔMoRAL tended to have a better predictive performance for OS than the ART score, which is a calculated index including liver function.

## 4. Material and Methods

### 4.1. Patients

Patients who had undergone TACE for treatment-naïve intermediate-stage HCC at Seoul National University Hospital (SNUH; Seoul, Korea) from January 2012 to June 2017 were eligible for this retrospective cohort study as a training cohort. The diagnosis of HCC was made by histology or dynamic imaging (computed tomography (CT) or magnetic resonance imaging (MRI) scans) [13,14]. Patients aged between 20 and 80 years at the time of the first TACE, who underwent at least two TACE sessions within 90 days, were included for analysis. All enrolled patients had no evidence of extrahepatic metastasis. Patients who had incomplete data of tumor markers or who received TACE as a bridge treatment for LT or for down-staging treatment before resection were excluded.

Two external validation cohorts were established: the hospital validation cohort and the nationwide validation cohort. The hospital validation cohort consisted of patients treated at the Samsung Medical Center (SMC; Seoul, Korea) and Ewha Womans University Medical Center (EUMC; Seoul, Korea) between February 2009 and December 2012, with the same inclusion/exclusion criteria. The nationwide validation cohort was acquired from the Korean Primary Liver Cancer Registry between 2008 and 2014, with the same inclusion/exclusion criteria (Supplementary Methods).

This study was conducted in accordance with the World Medical Association Declaration of Helsinki and was approved by the Institutional Review Board of each participating center (SNUH No. 1702-067-831, SMC No. 2018-08-137, and EUMC No. 2016-07-081-015), and by the Korean Central Cancer Registry, Ministry of Health and Welfare Korea, and the Korean Liver Cancer Association.

### 4.2. Treatment, Procedures and Assessments

Baseline laboratory and radiological findings of the enrolled patients in the training cohort and validation cohorts were assessed within seven days before the first TACE session. The MoRAL score was calculated as follows: 11×√PIVKA+2×√AFP [21]. To measure the changes in MoRAL score (ΔMoRAL), the MoRAL score was calculated at two time points. The baseline MoRAL score was calculated within seven days before the first TACE session. The second MoRAL score was recalculated one day before the second TACE session. The ΔMoRAL scores were found by comparing the second score with the baseline MoRAL score for each patient. We defined a MoRAL-increase when the second MoRAL score increased by ≥5% compared to the baseline MoRAL score, whereas a MoRAL-non-increase was defined by the second MoRAL score increasing by <5% or decreasing compared to the baseline MoRAL score.

Super selective TACE was performed by experienced interventional radiologists (>10 years of experience) at the participating centers according to tumor distribution and underlying liver function, as described elsewhere (Supplementary Methods) [33,34,35,36]. After a maximum of 90 days from the initial TACE session, dynamic imaging was performed to evaluate tumor response and repeated TACE was done when residual or newly developed tumors were observed without the presence of Child-Pugh C liver function or Eastern Cooperative Oncology Group performance status ≥2.

Radiological tumor response was assessed by dynamic CT or MRI scans prior to the second TACE session, according to the European Association for the Study of the Liver (EASL) criteria (Supplementary Methods) [37]. We defined partial response (PR) as the presence of a tumor response, and stable disease (SD) and progressive disease (PD) as the absence of a tumor response. Patients with complete response (CR) were not included in this study because they did not receive further TACE sessions after the first TACE within 90 days.

### 4.3. Endpoint

The primary endpoint was OS, which was measured from the day prior to the second TACE session until death from any cause or last follow-up. The survival data of the enrolled patients were obtained from the national statistical data from the Korean Ministry of Government Administration and Home Affairs. The data cutoff dates were 31 August 2018 in the training cohort and hospital validation cohort, and 31 December 2016 in the nationwide validation cohort.

### 4.4. Statistical Analysis

Kaplan-Meier curves were generated for OS and the log-rank test was used for group comparisons. A multivariable stepwise Cox regression analysis was performed to find independent predictors of OS. The Harrell concordance (*c*)-index was calculated to compare the discriminatory abilities of ΔMoRAL and other indices for predicting OS. The statistical analyses were performed using IBM SPSS Statistics for Windows, version 23.0 (IBM Corp., Armonk, NY) and R language, version 3.5.2 (R Foundation for Statistical Computing, Vienna, Austria). All statistical tests were 2-sided and *P* values less than 0.05 were considered statistically significant.

## 5. Conclusions

In conclusion, we have derived and validated the MoRAL score-based algorithm for predicting OS after the first TACE session for patients with intermediate-stage HCC, which is based on objective and reproducible predictors. This algorithm might provide refined prognostication for predicting OS after TACE. Specifically, patients with a high baseline MoRAL score (≥89.5) and MoRAL-increase between the first and second sessions of TACE had a dismal prognosis, and might be potential candidates for systemic therapy. Future clinical trials directly comparing repeated TACE and systemic therapy are warranted to verify the clinical efficacy of this algorithm.

## Figures and Tables

**Table 1:** Clinical characteristics in the three study cohorts.

Variables	Training Cohort (n = 193)	Hospital validation Cohort (n = 140)	Nationwide Validation Cohort (n = 149)
Before the first session of TACE			
Age	63.0 (55.0–70.0)	60.0 (53.0–68.0)	61.0 (54.0–68.0)
Male sex	164 (85.0%)	117 (83.6%)	127 (85.2%)
Etiology			
HBV infection	132 (68.4%)	99 (70.7%)	96 (64.4%)
HCV infection	24 (12.4%)	12 (8.6%)	20 (13.4%)
Alcohol	20 (10.4%)	20 (14.3%)	24 (16.1%)
Others	17 (8.8%)	9 (6.4%)	9 (6.0%)
Child-Pugh class			
A	164 (85%)	128 (91.4%)	125 (83.9%)
B 7 points	18 (9.3%)	9 (6.4%)	15 (10.1%)
≥B 8 points	11 (5.7%)	3 (2.1%)	9 (6.0%)
Tumor number	4.0 (2.0–5.0)	4.0 (2.0–5.0)	4.0 (2.0–5.0)
Maximum tumor size	5.0 (3.3–8.4)	4.6 (3.5–7.0)	5.0 (3.4–7.0)
AFP, ng/mL	38.8 (8.2–711.0)	29.0 (8.8–529.2)	59.6 (14.6–874.1)
PIVKA-II, mAU/mL	358.0 (48.0–3499.0)	288.0 (41.5–1200.0)	693.0 (88.0–2000.0)
MoRAL score	259.2 (89.5–780.6)	237.8 (93.1–419.3)	311.9 (133.0–544.6)
Before the second session of TACE			
AFP, ng/mL	15.2 (5.3–85.9)	14.4 (6.2–120.0)	–
PIVKA-II, mAU/mL	42 (24–236)	53.8 (22.0–450.0)	–
MoRAL score	93.8 (60.9–231.4)	103.2 (62.7–263.8)	–
Radiological tumor response ^a^			–
Absent (SD/PD)	55 (28.5%)	44 (31.4%)	
Present (PR)	138 (71.5%)	96 (68.6%)	
Dynamics of variables			
Child-Pugh increase			–
Absent	145 (75.1%)	108 (77.1%)	
+1 point	43 (22.3%)	30 (21.4%)	
+2 points or more	5 (2.6%)	2 (1.4%)	
AST increase >25%	22 (11.4%)	26 (18.6%)	–
ΔMoRAL			–
MoRAL-increase	33 (17.1%)	41 (29.3%)	
MoRAL-non-increase	163 (82.9%)	99 (70.7%)	

Abbreviations: TACE—transarterial chemoembolization; HBV—hepatitis B virus; HCV—hepatitis C virus; AFP—alpha-fetoprotein; PIVKA-II—protein induced by vitamin K absence-II; MoRAL—model to predict tumor recurrence after living donor liver transplantation; SD—stable disease; PD—progressive disease; PR—partial response; AST—aspartate aminotransferase. ^a^ Evaluated before the second session of TACE according to EASL criteria.

**Table 2:** Univariable analysis of prognostic factors in the training cohort (n = 193).

Variables	Group Analysis	Overall Survival (Months)			*p* Value ^a^
		n = 193	Median	95% CI	
Age	<65	109	37.8	26.9–45.6	
	≥65	84	32.5	26.9–42.9	0.685
Sex	Male	164	33.3	26.9–42.1	
	Female	29	44	29.9–61.5	0.331
Etiology	Viral	156	39.3	32.1–45.6	
	Other	37	26.9	19.7–33.5	0.150
Child–Pugh class	A	151	39.5	32.5–45.6	
	B	42	23.9	17.8–32.8	0.020
Tumor number	<4	86	40.2	28.4–61.5	
	≥4	107	32.8	26.6–39.5	0.086
Tumor size, cm	<5	95	40.2	27.5–46.6	
	≥5	98	32.8	26.9–39.5	0.847
AFP, ng/mL	<200	125	33.3	28.4–60.7	
	≥200	68	34.3	25.6–42.9	0.229
PIVKA–II, mAU/mL	<500	105	37.8	28.4–46.6	
	≥500	88	33.5	25.8–43.0	0.991
Baseline MoRAL score	<314.8	105	37.8	28.4–46.6	
	≥314.8	88	32.7	25.3–43.0	0.689
Radiological tumor response ^b^	Absent (SD/PD)	55	26.9	18.5–33.2	
	Present (PR)	138	40.2	31.8–46.6	0.014
AST increase >25%	Absent	171	37.4	31.8–43.0	
	Present	22	26.9	17.8–29.9	0.123
Child–Pugh increase	Absent	145	42.1	33.2–49.1	
	Present	48	26.9	24.0–32.1	0.001
ΔMoRAL	MoRAL-non-increase	160	37.8	31.8–45.6	
	MoRAL-increase	33	18.8	9.9–32.8	0.003

Abbreviations: AFP—alpha-fetoprotein; PIVKA-II—protein induced by vitamin K absence-II; MoRAL—model to predict tumor recurrence after living donor liver transplantation; SD—stable disease; PD—progressive disease; PR—partial response; AST—aspartate aminotransferase; TACE—transarterial chemoembolization; EASL—European Association for the Study of the Liver. ^a^ By log-rank test ^b^ Evaluated before the second session of TACE according to EASL criteria.

**Table 3:** Multivariable analysis of prognostic factors in the training cohort (*n* = 193).

Variables	Group Analysis	Model 1			Model 2		
		Overall Survival (Months)		*p* Value ^a^	Overall Survival (Months)		*p* Value ^a^
		Hazard Ratio	95% CI		Hazard Ratio	95% CI	
Child-Pugh class	A	1 (Ref)	–	–	1 (Ref)	–	–
	B	1.57	1.02–2.42	0.039	1.59	1.04–2.45	0.034
Child-Pugh score increase	Absent	1 (Ref)	–	–	1 (Ref)	–	–
	Present	1.84	1.23–2.76	0.003	2.03	1.35–3.05	0.001
Radiological tumor response ^b^	Present (PR)	1 (Ref)	–	–	–	–	–
	Absent (SD/PD)	1.63	1.08–2.46	0.019			
ΔMoRAL	MoRAL-non-increase	–	–	–	1 (Ref)	–	–
	MoRAL-increase				2.18	1.37–3.46	0.001

Abbreviations: SD—stable disease; PD—progressive disease; PR—partial response; MoRAL—model to predict tumor recurrence after living donor liver transplantation. ^a^ By Cox proportional hazard model ^b^ Evaluated before the second session of TACE according to EASL criteria.

**Table 4:** Comparison between ΔMoRAL and other models for predicting overall survival.

Prediction Models	Training Cohort	Hospital Validation Cohort
ΔMoRAL		
*c*-index (95% CI)	0.73 (0.62–0.84)	0.72 (0.62–0.83)
Baseline MoRAL score		
*c*-index (95% CI)	0.55 (0.48–0.61)	0.59 (0.53–0.66)
*p* ^a^	0.005	0.04
Baseline AFP		
*c*-index (95% CI)	0.55 (0.49–0.61)	0.59 (0.53–0.66)
*p* ^a^	0.005	0.04
Baseline PIVKA-II		
*c*-index (95% CI)	0.52 (0.46–0.59)	0.59 (0.52–0.66)
*p* ^a^	0.002	0.03
AFP increase		
*c*-index (95% CI)	0.60 (0.47–0.74)	0.64 (0.51–0.77)
*p* ^a^	0.04	0.17
PIVKA-II increase		
*c*-index (95% CI)	0.66 (0.53–0.79)	0.55 (0.41–0.69)
*p* ^a^	0.02	0.002
Radiological tumor response ^b^		
*c*-index (95% CI)	0.67 (0.57–0.77)	0.64 (0.51–0.76)
*p* ^a^	0.20	0.13
ART score of 2.5	
*c*-index (95% CI)	0.68 (0.57–0.79)	0.61 (0.49–0.73)
*p* ^a^	0.24	0.07
ART score + ΔMoRAL of 2.5	
*c*-index (95% CI)	0.73 (0.63–0.84)	0.64 (0.52–0.75)
*p* ^a^	0.52	0.09

Abbreviations: MoRAL—model to predict tumor recurrence after living donor liver transplantation; AFP—alpha-fetoprotein; PIVKA-II—protein induced by vitamin K absence-II. ^a^ Compared to the *c*-index of ΔMoRAL ^b^ Evaluated before the second session of TACE according to EASL criteria.

## Supplementary Materials

The following are available online at https://www.mdpi.com/2072-6694/11/11/1721/s1, Figure S1: CONSORT diagrams of the training cohort; Figure S2: Correlation between each radiologic tumor response and the ΔMoRAL percentage; Figure S3: Prognostic significance of the baseline MoRAL score of 314.8 in the hospital validation cohort. Table S1: Multivariable analysis of prognostic factors in the training cohort excluding patients with alcoholic liver disease (n = 173).
