# Supplementary material for: Tumor Marker-Based Definition of the Transarterial Chemoembolization-Refractoriness in Intermediate-Stage Hepatocellular Carcinoma: A Multi-Cohort Study
Source: Cancers (Basel). 2019 Nov 4;11(11):1721. doi: 10.3390/cancers11111721 (PMC6896068; doi:10.3390/cancers11111721)
Supplement: Supplementary file 1 [file cancers-11-01721-s001.pdf]

cancers MDPI

Tumor Marker-Based Definition of the Transarterial Chemoembolization-Refractoriness in Intermediate-Stage Hepatocellular Carcinoma: A Multi-Cohort Study

Jun Sik Yoon, Dong Hyun Sinn, Jeong-Hoon Lee, Hwi Young Kim, Cheol-Hyung Lee, Sun Woong Kim, Hyo Young Lee, Joon Yeul Nam, Young Chang, Yun Bin Lee, Eun Ju Cho, Su Jong Yu, Hyo-Cheol Kim, Jin Wook Chung, Yoon Jun Kim and Jung-Hwan Yoon

Supplemental Methods

Patients

The Korean Primary Liver Cancer Registry is a random sample of approximately 15% of newly diagnosed HCC patients from the Korea Central Cancer Registry, which is a nationwide cancer registry and includes more than 95% of all cancer cases in Korea. The database contains patient' information including demographics, etiology, Child-Pugh class, tumor number, maximum tumor size, baseline laboratory findings, BCLC stage, treatment modality, dates of first and second treatments, and survival outcomes.

Treatment Procedures and Assessments

During TACE, according to the Seldinger technique, an arterial catheter was inserted into the femoral artery and angiography of the superior mesenteric, celiac, and common hepatic arteries was performed. The catheter was then advanced into the hepatic artery branch and positioned into or as close as possible to the tumor-feeding branch. TACE was performed using a mixture of 2 to 20 mL of iodized oil (Lipiodol; Guerbet, Aulnay-sous-Bois, France) and 20 to 60 mg of doxorubicin hydrochloride (ADM; Dong-A Pharmacy, Seoul, Korea). Embolization of the tumor-feeding artery using absorbable gelatin sponge particles (Gelfoam; Upjohn, Kalamazoo, MI, USA) was performed until stasis in the segmental or subsegmental arterial branches was achieved. Patients were admitted to the hospital on the day before the procedure for hydration and were discharged within 24 hours after TACE if there were no immediate complications.

We defined radiological tumor response as follows: partial response (PR; >50% reduction in total tumor load of all measurable lesions); stable disease (SD; disease not qualifying as complete response [CR]/PR or progressive disease (PD)); PD (>25% increase in size of 1 or more measurable lesions or the appearance of new lesions).

Supplemental Figures

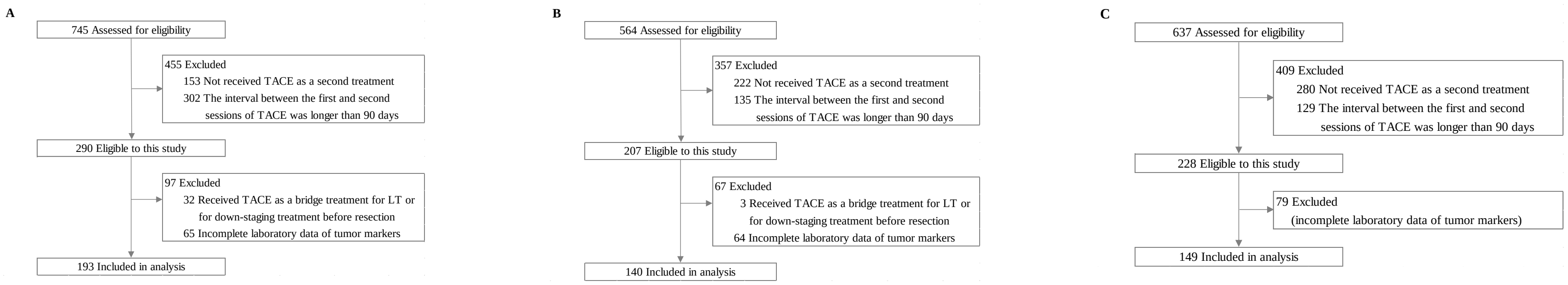

Figure S1. CONSORT diagrams of the training cohort (**A**), the hospital validation cohort (**B**), and the national validation cohort (**C**). Abbreviations: TACE, transarterial chemoembolization; LT, liver transplantation.

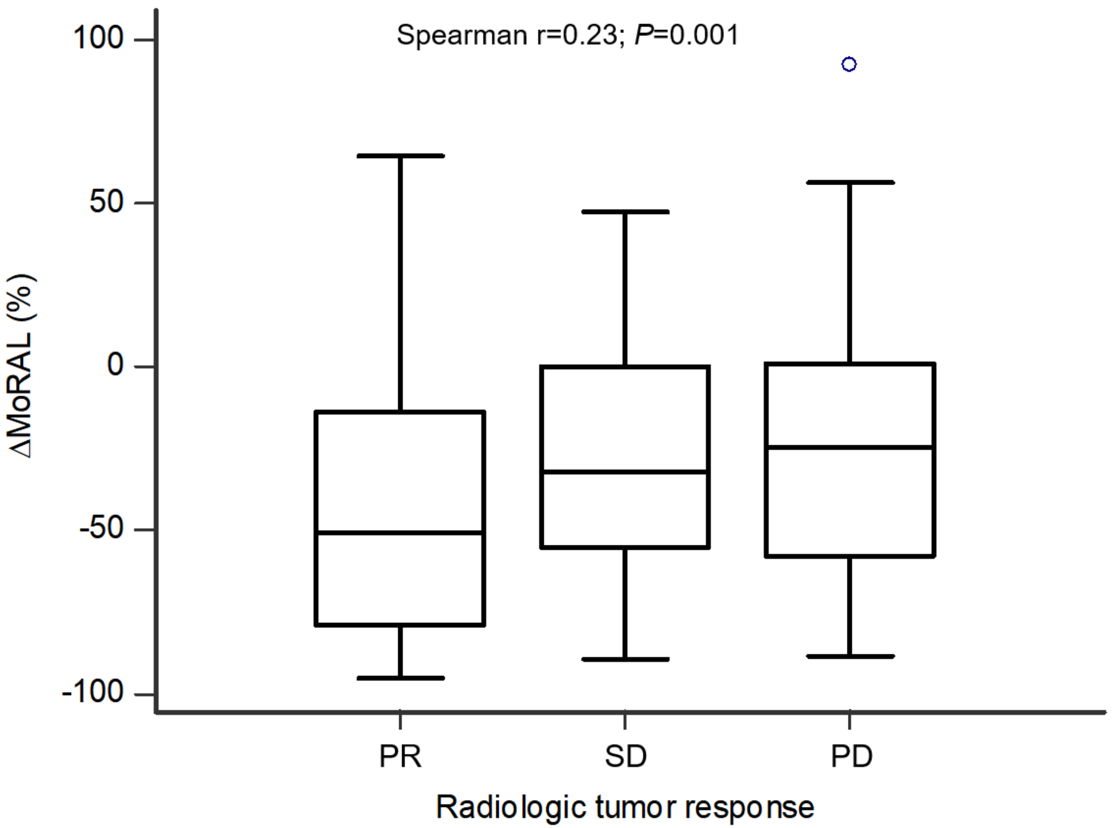

Figure S2. Correlation between each radiologic tumor response and the ΔMoRAL percentage. Abbreviations: PR, partial response; SD, stable disease; PD, progressive disease.

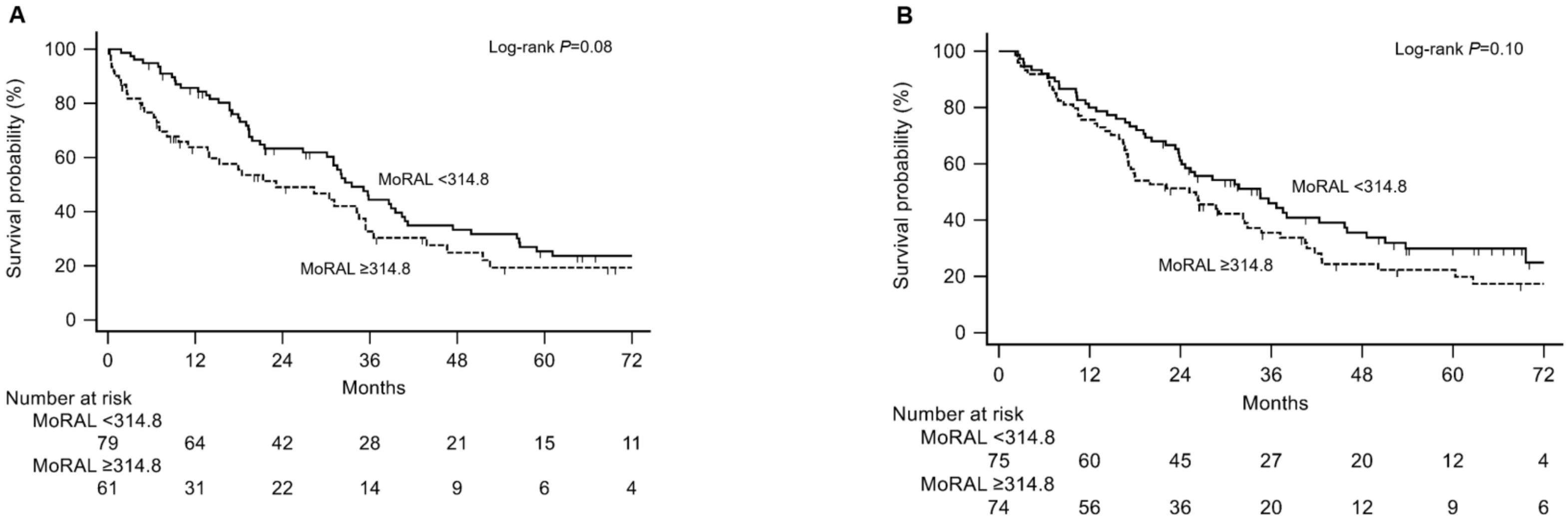

Figure S3. Prognostic significance of the baseline MoRAL score of 314.8 in the hospital validation cohort (**A**) and nationwide validation cohort (**B**).

Table S1. Multivariable analysis of prognostic factors in the training cohort excluding patients with alcoholic liver disease (n = 173).

		**Model 1**			**Model 2**		
	Variables	Overall survival (Months) Hazard ratio	95% CI	*p* value[a]	Overall survival (Months) Hazard ratio	95% CI	*P* value[a]
Child-Pugh class	A	1 (Ref)	–	–	1 (Ref)	–	–
	B	1.58	1.02–2.46	0.043	1.60	1.03–2.50	0.037
Child-Pugh score increase	Absent	1 (Ref)	–	–	1 (Ref)	–	–
	Present	1.71	1.12–2.62	0.013	1.89	1.23–2.91	0.004
Radiological tumor response[b]	Present (PR)	1 (Ref)	–	–	–	–	–
	Absent (SD/PD)	1.59	1.04–2.44	0.032			
ΔMoRAL	MoRAL-non-increase	–	–	–	1 (Ref)	–	–
	MoRAL-increase				2.06	1.27–3.35	0.004

Abbreviations: SD, stable disease; PD, progressive disease; PR, partial response; MoRAL, model to predict tumor recurrence after living donor liver transplantation.
[a] By Cox proportional hazard model [b] Evaluated before the second session of TACE according to EASL criteria.
